# Inclusive community playgrounds benefit typically developing children: An objective analysis of physical activity

**DOI:** 10.3389/fspor.2022.1100574

**Published:** 2023-02-01

**Authors:** Wilshaw R. Stevens, Justine M. Borchard, Paige Sleeper, Dana Dempsey, Kelly A. Jeans, Chan-Hee Jo, Kirsten Tulchin-Francis

**Affiliations:** ^1^ Movement Science Lab, Scottish Rite for Children, Dallas, TX, United States; ^2^ Therapeutic Recreation, Scottish Rite for Children, Dallas, TX, United States; ^3^ Research Department, Scottish Rite for Children, Dallas, TX, United States; ^4^ Department of Orthopedic Surgery, Nationwide Children’s Hospital, Columbus, OH, United States

**Keywords:** inclusive, playground, community, physical activity, intensity/duration, heart rate

## Abstract

**Purpose:**

Limited research is available on the physical activity levels of children while playing on an inclusive playground, specifically designed to accommodate children with physical disabilities. The aims of this study were to objectively measure ambulatory activity and heart rate (HR) of children during unstructured play on an inclusive community playground.

**Methods:**

Typically developing children at least 4 years of age were recruited to play freely upon entering the playground. Participants wore a StepWatch4 Activity Monitor and a Polar V800 Sport Watch. Ambulatory measures included total steps, percentage of recommended steps, total ambulatory time (TAT), bout intensity levels/duration periods. Time spent in HR zones and moderate-to-vigorous physical activity (MVPA) was determined.

**Results:**

95 children (48 males; Avg. age: 7 ± 2 years.) were included in this study. Children played for 31.8 ± 14.7 min., were ambulatory for 25.9 ± 12.0 min., took 1826 ± 824 steps, and accumulated 17 ± 8% of the recommended daily step count. Ambulatory bout intensity was predominantly lower intensity and bout durations varied in length. 99% of the play time was spent at a moderate HR or higher. Significant correlations were found between ambulatory and HR measures (*ρ* range from 0.23 to 0.99, *p* < 0.05), and *7–10 yo* children spent a significantly higher percentage of TAT at higher intensity ambulation (*p* < 0.05).

**Conclusions:**

Typically developing children can achieve moderate or higher intensity exercise and HR on an inclusive playground. Both typically developing children and those with disabilities, would benefit from a setting where they can interact and participate in parallel play with their peers.

## Introduction

A recent global study on children showed that more than 80% do not meet physical activity (PA) guidelines (1). The World Health Organization (WHO) has developed a global strategy on diet, health and PA^1^. This plan outlines several ways to increase PA within communities and it is recommended that children and youth accumulate at least 60 min of moderate-to vigorous-intensity PA daily.

The 2018 United States PA Guidelines Advisory Committee Report demonstrates that higher amounts of PA are associated with reduced risk for an excessive weight gain in children 3–6 years of age (2). Greater emphasis should be placed on promoting PA in children, and researchers have been investigating the most effective strategies (3). There is evidence showing that access to indoor or outdoor recreation facilities is positively associated with greater PA among both children and adults (3, 4).

Playgrounds have been shown to be an effective resource promoting PA in children. Observational assessments of children on the playground have been conducted utilizing various tools (5–8). With advancements in wearable technology which can combine the measurement of ambulatory activity and heart rate (HR), there is great value in objectively measuring PA levels of children during unstructured playground-play. Studies utilizing such technology have shown that PA levels are influenced by environmental factors, differences in playground designs, sex and age (8–16). There is, however, limited research in the community playground setting (12–14).

City planners are also taking the necessary steps to construct playgrounds that are suitable for children with disabilities. These “inclusive playgrounds” allow children with disabilities to receive the health and psychosocial benefits of unstructured play along-side their typically developing peers (17). There is a negative perception that inclusive playgrounds cost significantly more and limit the typically developing child's ability to meet PA guidelines. An objective analysis of the PA of typically developing children on an inclusive playground has largely been unstudied. As more facilities are adapted and constructed for children of all physical abilities, it is important to understand, on a baseline level, the amount of physiological (elevated HR) and mechanical (ambulatory activity) loading the typically developing child experiences in these settings.

The purpose of this project was to objectively measure ambulatory activity and HR during unstructured play in typically developing children while playing on an inclusive community playground. It was hypothesized that typically developing children would spend significant portions of their time in play at a MVPA level, and the intensity/duration of ambulatory bouts would be predominantly high intensity for shorter duration periods. In addition, the relationships between age and overall play measures, such as total time spent on the playground, steps taken, and ambulatory time, to HR measures were explored.

## Methods

In this institutional review board approved study, typically developing children at least 4 years of age were recruited as they entered the community playground at Scottish Rite for Children, Dallas Campus. Subject recruitment and data collection (during daylight hours) were conducted over two summers, including June-August, 2018 and May–July, 2019. Pediatric patients of the hospital, who were diagnosed with a neurologic condition, and/or were treated for an orthopedic condition in the last year, were excluded. Therefore a convenience sample of typically developing children utilizing the community playground were invited to participate. Written consent was obtained from the parent or guardian, and assent was obtained from children over the age of 10 years.

The inclusive community playground, occupies approximately 6,000 square feet (557 square meters) and is open to the community all year round during the day, excluding days where the ground surface or playground equipment is wet. The playground is structured with traditional features that have been adapted for children with physical disabilities, who may utilize walking aids such as crutches, walkers or ankle-foot orthoses or who may be non-ambulatory (wheelchair). The playground surface is a semi-firm foam material that can be easily traversed by those using a walker or wheelchair.

The playground includes equipment for children ages 2–5 and 6–10 years. Features within the large play structures comprise of balance beams, sound features (i.e., bells, megaphones), climbing walls, slides and kinetic energy features (powered as the child quickly turns the handle producing various sounds). Coupled with these large play structures, include free-standing features for climbing/bouncing, sitting/spinning and a playhouse for imaginative play. Two unique play structures include the AeroGlider™ (Playworld®, Lewisburg, PA, United States), a large see-saw structure with a rocker-style platform, and the NEOS 360™ (Playworld®, Lewisburg, PA, United States), an interactive device where various competitive games can be performed.

Height, weight, age and sex were recorded, and the participant was fitted with a StepWatch4 Activity Monitor (SAM, Modus Health, Edmonds, WA, United States), to record ambulatory activity. The SAM is a research grade ambulatory activity monitor that provides a time stamp of when strides are taken (18). The SAM has been shown to be accurate and reliable for assessing the ambulatory activity of able-bodied children (19) and has been utilized to assess the intensity and duration of daily ambulatory bouts (20, 21). The SAM was worn on the lateral ankle just superior to the lateral malleolus, and the movement calibration settings were based on the participant's height which was entered into the software. To assess HR and overall distance traveled, a Polar V800 Sport Watch (Polar, Polar Electro Inc., Bethpage, NY, United States) with GPS capability along with a Polar H10 chest strap, were worn.

The SAM and Polar monitors were verified to be configured correctly, and GPS signal was obtained on the Polar watch. The research team confirmed with the participant that the comfort and fit of the devices was satisfactory, and the participant was instructed to play freely. Once the participant was ready to leave the playground, data collection concluded, and the devices were removed.

The raw SAM stride data were exported in Microsoft Excel file format along with a time stamp of the number of strides taken every 10 s. The Polar watch data was uploaded to the Polar Flow app, and a Microsoft Excel file containing the HR data and distance traveled every second was exported. A custom written MATLAB code (Mathworks, Natick, MA, United States) down-sampled the Polar data to the maximum HR every 10 s and synchronized it with the time stamp of the SAM output.

Ambulatory measures were calculated using previously published methods (20, 21). Single strides counted by the SAM were doubled to account for the other leg, and a total number of steps (strides × 2) was determined for each 10 s interval. The total steps taken during play (Steps) were summated and divided by 11,000 steps for a general estimate of the daily recommended step count for children (% of recommended steps) (22, 23). Steps in sequential intervals were grouped into ambulatory bouts (24). Total ambulatory time (TAT) in minutes was computed by summating the duration of all ambulatory bouts.

The intensity and duration of each individual ambulatory bout was calculated (20). Intensity levels were defined using the average cadence (steps/min) of typically developing age-matched children during over-ground walking at a self-selected speed, with definitions of *Easy* (<60% of cadence) and *Moderate+* (≥60% of cadence) (20). Duration of ambulatory bouts was calculated in minutes and summarized into the following categories: *Short* – less than 2 min, *Intermediate*—2–5 min, and *Long*—greater than 5 min. In addition, there were six intensity/duration combination categories for each ambulatory bout: *Easy/Short, Easy/Intermediate, Easy/Long, Moderate+/Short, Moderate+/Intermediate and Moderate+/Long*. The intensity/duration combination categories were reported as a percentage of the TAT.

*P*articipant maximum HR was calculated using a child specific regression equation based on their age (*208–0.7 ×  age*) (25). Due to the lack of research in younger children, this equation (previously validated for older children) was applied. HR zones were calculated as a percentage of each child's age-based, calculated maximum HR: *HR Easy* (<50% of max HR)*, HR Moderate* (50%–70% of max HR)*, HR Vigorous* (70- 85% of max HR) and *HR Peak* (>85% of max HR)^2^. The percentage of time in each HR zone was calculated along with the total number of minutes in each zone. The total number of minutes in MVPA was summed. Additional measures included in this analysis were outside temperature in degrees Fahrenheit (°F)/Celsius(°C), total distance traveled in meters (Distance, m) and total time playing (Total Time) in minutes.

Spearman's rank correlations (*ρ*) were made on all measures. Participants were also divided into three groups: *4–6 yo*, *7–10 yo* and *11+ yo*. A Mann-Whitney U test for nonparametric comparisons were run across the age groups and between sex. Statistical analysis was conducted using SPSS (version 24, IBM Inc., Chicago, IL, USA) Statistical significance was set at alpha 0.05.

## Results

There were 100 typically developing children who enrolled in this study. Two children were excluded from the data analysis due to a lack of documented written assent. Three children were excluded to a HR monitor hardware malfunction. There were no incidences of reported discomfort or refusal to wear the monitors.

For the 95 remaining children (Table 1), the average age was 7 ± 2 years old (4–12 years old). Due to the small number of participants in the 11+ yo cohort, this group was not included in the statistical analysis (Supplementary Figure S1 and Table S1). All statistical results presented in this paper included 88 children categorized into 4–6 yo and 7–10 yo age groups.

**Table 1:** Demographics; BMI—body mass Index; mean ± SD.

	Age Group			
	All Participants	*4–6 yo*	*7–10 yo*	*11+ yo*
Participant Count	95	41	47	7
Sex (Males/Females)	48/47	24/17	20/27	4/3
Age (years)	7 ± 2	5 ± 1	8 ± 1	12 ± 1
Height (m)	1.2 ± 0.1	1.1 ± 0.1	1.3 ± 0.1	1.5 ± 0.1
Weight (kg)	27 ± 9	22 ± 5	30 ± 8	41 ± 10
BMI (kg/m^2^)	17 ± 3	17 ± 2	17 ± 3	19 ± 2

There was no statistical difference in BMI between the age groups (*p* = 0.52). The majority of the children (86 of the 88 children, 98%) were observed playing with a sibling or friend during the data collection period. The average outdoor temperature on the day of testing was 88 ± 5°F (31 ± 3°C) and was not statistically significant between age groups (Table 2). The average total time spent playing was 31.5 ± 14.6 min (min.) and was not statistically different between the age groups (*4–6 yo* vs. *7–10 yo*, Table 2). An average of 1810 ± 822 steps were taken and TAT was 25.7 ± 12.1 min across the entire cohort. Neither were statistically significant between age groups (*p* > 0.05, Table 2). The play period accounted for 17 ± 8% of the recommended daily step count for both groups, and there was no statistically significant difference between age groups. Distance traveled as determined by the GPS was 518 ± 296 meters and was not significantly different between age groups (Table 2).

**Table 2:** Age group comparisons – overall play measures (mean ± SD).

	Age Group				
	Overall	*4–6 yo*	*7–10 yo*	*p - value*	*11+ yo* ^a^
	(*N* = 88)	(*N* = 41)	(*N* = 47)	* *	(*N* = 7)
Outside Temperature (°F)/(°C)	88 ± 5/31 ± 3	87 ± 5/31 ± 3	88 ± 5/31 ± 3	0.46	90 ± 4/33 ± 3
Total Time (min.)	31.5 ± 14.6	30.9 ± 12.8	31.6 ± 16.0	0.68	38.4 ± 16.6
Steps^b^	1810 ± 822	1769 ± 778	1829 ± 863	0.97	2134 ± 871
% of Recommended Steps (%)	17 ± 8	16 ± 7	17 ± 8	0.97	19 ± 8
TAT (min.)^c^	25.7 ± 12.1	24.9 ± 10.7	26.2 ± 13.0	0.68	29.7 ± 13.7
Distance (m)	518 ± 296	506 ± 301	524 ± 291	0.80	699 ± 239

^a^ *11+ yo* participants listed here for descriptive purposes only and are not included in statistical analysis.

^b^ Steps – total steps taken.

^c^ TAT – total ambulatory time (minutes).

Ambulatory bout intensity levels were summarized into *Easy* or *Moderate+* and were reported as a percentage of TAT. The percentage of TAT at *Easy* intensity was significantly higher in *4–6 yo* compared to *7–10 yo* (Supplementary Figure S2A). *7–10 yo* group spent a significantly greater percentage of TAT at *Moderate+* intensity compared to the *4–6 yo* group. Ambulatory bout duration periods were summarized into *Short*, *Intermediate* or *Long*, reported as a percentage of TAT. There were no differences between age groups in *Short* (*p* = 0.39), *Intermediate* (*p* = 0.62) or *Long* duration ambulatory bouts (*p* = 0.29) (Supplementary Figure S2B).

Significant differences between the age groups were seen in the combined intensity/ duration categories (Table 3). There were no significant differences between age groups in the percentage of ambulatory time in the *Easy/Short*, *Easy/Intermediate* or *Easy/Long* categories (Table 3). *Moderate+/Intermediate* ambulatory activity was significantly higher in the *7–10 yo* group. This significant difference was also observed in *Moderate+/Long* ambulatory activity, as the *7–10 yo* group spent more ambulatory time. There was no significant difference between groups in *Moderate+/Short* ambulatory activity (Table 3).

**Table 3:** Age group comparisons – ambulatory and heart rate measures (mean ± SD; *p-value <0.05).

	Age Group				
	Overall	*4–6 yo*	*7–10 yo*	*p-*value	*11+ yo* ^a^
Ambulatory Intensity/Duration^b^					
*Easy/Short* (%)	26.8 ± 17.1	29.5 ± 15.6	25.1 ± 18.0	0.12	22.2 ± 18.9
*Easy/Intermediate* (%)	30.9 ± 21.0	35.4 ± 22.3	29.5 ± 19.3	0.16	13.5 ± 14.2
*Easy/Long* (%)	19.2 ± 24.6	20.5 ± 25.9	19.7 ± 24.6	0.93	9.1 ± 15.5
*Moderate+/Short* (%)	5.8 ± 7.2	4.5 ± 7.0	5.6 ± 5.0	0.08	15.5 ± 12.8
***Moderate+/Intermediate* (%)**	9.7 ± 14.6	**6.1 **± **14.2**	**10.6 **± **12.1**	**<0**.01*	24.4 ± 23.2
***Moderate+/Long* (%)**	7.6 ± 17.4	**4.0 **± **15.5**	**9.6 **± **17.9**	**0**.03*	15.2 ± 23.2
Heart Rate^c^					
*HR Easy* (%)	0.8 ± 3.0	1.1 ± 4.3	0.5 ± 1.5	0.56	0.1 ± 0.3
*HR Moderate* (%)	26.9 ± 21.7	24.3 ± 21.0	30.9 ± 22.7	0.19	15.1 ± 12.3
*HR Vigorous* (%)	51.3 ± 16.4	52.5 ± 16.6	50.0 ± 16.0	0.42	53.9 ± 20.5
*HR Peak* (%)	21.0 ± 19.2	22.0 ± 19.2	18.7 ± 18.2	0.54	30.9 ± 24.2
*HR Easy* (min.)	0.2 ± 0.9	0.4 ± 1.3	0.2 ± 0.4	0.56	0.1 ± 0.2
*HR Moderate* (min.)	8.9 ± 9.0	7.3 ± 6.9	10.8 ± 10.7	0.25	5.9 ± 6.0
*HR Vigorous* (min.)	16.6 ± 10.3	16.7 ± 10.0	15.7 ± 9.4	0.42	22.5 ± 16.9
*HR Peak* (min.)	6.2 ± 5.8	6.7 ± 6.3	5.2 ± 4.7	0.52	10.0 ± 7.7
MVPA (min.)^d^	31.7 ± 14.7	30.7 ± 12.7	31.6 ± 16.0	0.75	38.4 ± 16.5

^a^ *11+ yo* participants listed here for descriptive purposes only and are not included in statistical analysis.

^b^ Ambulatory Intensity Levels/Duration periods reported as a percentage of total ambulatory time (TAT).

^c^ Heart Rate (HR) zones reported as a percentage of maximum age-based HR and reported in number of minutes in each zone.

^d^ MVPA – moderate-to-vigorous (including peak) physical activity.

The HR data, as previously described, was categorized into the following zones based on a percentage of age-based calculated maximum HR: *HR Easy*, *HR Moderate*, *HR Vigorous* and *HR Peak*. There were no significant differences seen in the percentage time spent in any of the *HR* categories (*p* > 0.05, Table 3). Only a very small percentage of time was spent in the *HR Easy* category (0.8 ± 3.0%, all groups mean), with approximately 77% of the time spent at *HR Moderate and HR Vigorous combined* and 21.0 ± 19.2% at *HR Peak*. The total number of minutes of MVPA was 31.7 ± 14.7 min., and this was not significantly different between age groups (Table 3).

Across all ambulatory and HR measures, there were no significant differences between sex within each age group (*4–6 yo* females vs. *4–6 yo* males, *7–10 yo* females vs. *7–10 yo* males, *p* > 0.05). There were statistically significant differences when comparing the same sex between each age group (*4–6 yo* females vs. *7–10 yo* females and *4–6 yo* males vs. *7–10 yo* males, Supplementary Table S2). In *Moderate+/Intermediate*, *7–10 yo* males spent a higher percentage of ambulatory time when compared to *4–6 yo* males. There was a trend for Easy*/Short* and *Easy/Intermediate* being significantly higher in *4–6 yo* males compared to *7–10 yo* males (*p* = 0.06). The amount of time in *HR Moderate* was significantly higher in *7–10 yo* males (Supplementary Table S2).

When comparing *4–6 yo* females to 7*–10 yo* females, significant differences were observed only in the ambulatory activity measures. The amount of time spent in *Easy* intensity was significantly higher in *4–6 yo* females. This difference became even more apparent when assessing the amount of time spent in *Moderate+/Long* as *4–6 yo* females spent no ambulatory in this category (Supplementary Table S2).

**Figure 1:**
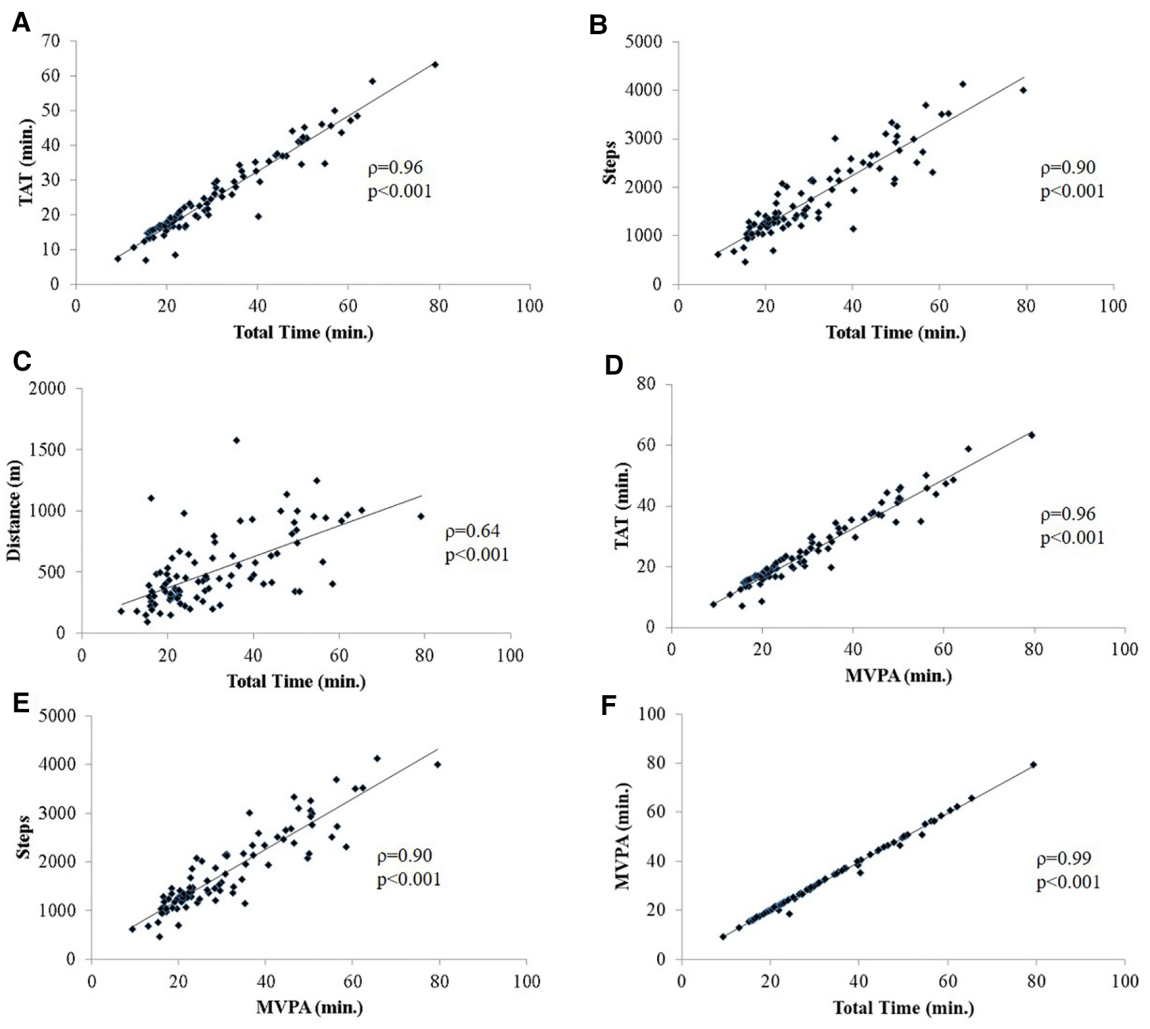
(**A–F**). Spearman rank correlations (*ρ*) between overall play measures; (**A**) total time spent on the playground (Total Time, minutes) and total ambulatory time (TAT, minutes), (**B**) total steps taken (Steps), (**C**) total distance traveled in meters (Distance, m); (**D**) minutes of moderate-to-vigorous (including peak) physical activity (MVPA, min.) and TAT, (**E**) Steps; (**F**) Total Time; all correlations were run including 4–6 yo and 7–10 yo participants only. *p*-value <0.05.

There was a strong positive correlation between the total time that subject's spent on the playground (Total Time) with their TAT (*ρ* = 0.96, *p* < 0.01, Figure 1A), the number of steps taken (Steps, *ρ* = 0.90, *p* < 0.01, Figure 1B) and distance traveled (Distance, *ρ* = 0.64, *p* < 0.01, Figure 1C). Total minutes of MVPA was positively correlated to TAT (*ρ* = 0.96, *p* < 0.01, Figure 1D), Steps (*ρ* = 0.90, *p* < 0.01, Figure 1E) and Total Time (*ρ* = 0.99, *p* < 0.01, Figure 1F). A weak negative correlation was observed between outside temperature and Distance (*ρ* = −0.25, *p* = 0.01, Supplementary Figure S3A). Distance was positively correlated to Steps (*ρ* = 0.76, *p* < 0.01, Supplementary Figure S3B). Total minutes of MVPA was also positively correlated to Distance (*ρ* = 0.62, *p* < 0.01, Supplementary Figure S3C).

There was a weak negative correlation between age and the intensity/duration categories of *Easy/Short* (*ρ* = −0.23, *p* = 0.03) and *Easy/Intermediate* (*ρ* = −0.21, *p* = 0.04) (Supplementary Figures S4A,B). There was a weak positive correlation between age and the combination categories of *Moderate+/Short* (*ρ* = 0.25, *p* = 0.02), *Moderate+/Intermediate* (*ρ* = 0.33, *p* < 0.01) and *Moderate+/Long* (*ρ* = 0.38, *p* < 0.01) (Supplementary Figures S4C–E).

There was a positive correlation between Total Time and minutes in the following HR zones: *HR Moderate* (*ρ* = 0.44, *p* < 0.01), *HR Vigorous* (*ρ* = 0.79, *p* < 0.01), and *HR Peak* (*ρ* = 0.23, *p* = 0.02) (Supplementary Figures S5A–C). The total steps taken (Steps) was positively correlated to minutes at *HR Moderate* (*ρ* = 0.30, *p* < 0.01), *HR Vigorous* (*ρ* = 0.73, *p* < 0.01,), and *HR Peak* (*ρ* = 0.29, *p* < 0.01) (Supplementary Figures S5D–F). TAT was positively correlated to minutes at *HR Moderate* (*ρ* = 0.41, *p* < 0.01), *HR Vigorous* (*ρ* = 0.79, *p* < 0.01,), and *HR Peak* (*ρ* = 0.25, *p* = 0.02) (Supplementary Figures S5G–I). Distance was positively correlated to minutes at *HR Vigorous* (*ρ* = 0.47, *p* < 0.01), and *HR Peak* (*ρ* = 0.39, *p* < 0.01).

## Discussion

The current study utilized wearable activity monitors to objectively measure ambulatory activity and HR of typically developing children during unstructured play in an inclusive community playground. Research on PA patterns of typically developing children on a community playground designed for varying levels of physical disabilities is largely understudied. It was feasible to utilize the SAM to measure ambulatory activity and a Polar HR monitor to assess time spent in specific HR zones. These different methodological approaches allowed for an in-depth analysis of the level of activity performed, and researcher's utilizing such wearable technologies can observe the advantages to both.

In this study, children spent 30 min on the playground with most of the time (∼80% of total time on the playground) spent in active ambulation. By using total steps taken as a global assessment of ambulatory activity, it was revealed that playground play accounted for more than 16% of the daily step count recommendation. Though daily step counts have been shown to be quite variable in children (22, 23), it is encouraging to observe that a reasonable amount of ambulatory activity occurred in a relatively short period of time. The present study evaluates ambulatory activity by “ambulatory bout”, which allows for a more discrete analysis of the *quality* of the activity (20, 24).

The evaluation of objectively measured intensity/duration during ambulatory activity in an inclusive community playground setting has not been previously reported. The predominant duration period of ambulatory activity was 2–5 min (*Intermediate*) and was not significantly different between *4 and 6 yo* and *7–10 yo* children. Play patterns varied in bout duration for both age groups as there was an uneven distribution in the amount of ambulatory time spent across the duration periods. Contrary to our hypothesis, ambulatory bout intensity was not predominantly at the higher intensity level *Moderate+*. Age group analysis showed that *7–10 yo* children engaged in more *Moderate+/Intermediate* and *Moderate+/Long* ambulatory activity (Table 3), and correlations revealed positive weak relationships between increasing age and a higher percentage of time spent at *Moderate+* ambulatory activity (Supplementary Figures S4C–E). This suggests an added health benefit for the older children, who may reach higher intensity activity levels while engaged in playground play.

The United States PA Guidelines Advisory Committee most recently reported that there is a paradigm shift in public health recommendations to support greater emphasis on total daily MVPA as an important lifestyle behavior regardless of the bout duration (26). The WHO PA guidelines for children and youth also state that efforts to accumulate 60 min of MVPA can be obtained by performing multiple shorter bouts spread throughout the day^1^. Our findings showed that children spent almost all of their time in MVPA while on the playground (∼99%, more than 30 min). Overall, there was a strong positive correlation between MVPA and Total Time, Steps and TAT (*ρ* > 0.90) (Figure 1). Fjørtoft et al. also showed that children 6 years of age achieved a moderate HR or higher for more than 20 min while playing on a typical playground (10). In a large study assessing the impact of a playground redesign, children (average age: 8 years) spent between 24%–37% of their recess time in MVPA. Similar to our study, the amount of MPVA increased with playtime duration (16). Unique to the Ridgers et al. study was the fact that playgrounds were redesigned into zones which also included a quiet play zone, which may explain the lower percentage of time in MVPA and highlight the fact that play structures can have an influence on PA levels (16). Adams et al. showed that children spent between 10 and 14 min in MVPA across various community playgrounds designs, however, activity levels were categorized by hip-worn accelerometers (12).

Researchers have reported age and sex differences in PA on the playground with boys reported to have engaged in more MVPA (27, 28). In the present study, there were no statistically significant sex related differences (*p* > 0.05) observed within the age groups (e.g., *4–6 yo* males vs. *4–6 yo females*), however, there were differences between the age groups when separated by sex (e.g., *4–6 yo males* vs. *7–10 yo males*). Older 7*–10 yo* males demonstrated twice the time in the *HR Moderate* zone when compared to younger male children (Supplementary Table S2). In females, *4–6 yo* females did not spend any time in Moderate*+/Long* ambulatory activity when compared to almost 10% TAT in older female children (Supplementary Table S2). Though not specifically assessed only during playground play, Tulchin-Francis et al. showed that children 10 years and older spent more ambulatory time throughout the day in *Moderate+/Intermediate* and *Moderate+/Long* compared to younger children (ages seven to 9 years) (20). These findings, in conjunction with the current study, suggest older children are able to sustain continuous play longer than younger children, which may be related to multiple factors (attention span, social skills, peer interaction). Future research is needed to investigate the complexity of playground play (playground features, social interaction, etc.).

The shift from strictly observational studies of playground play towards studies that also obtain objective measures using a wearable device (accelerometers, HR monitors, etc.), has been emerging in recent years (29). McCrorie et al. highlights the need for more longitudinal studies on objectively measured PA of children (30). While an ambulatory activity monitor measures step accumulation there is additional useful information provided by the HR data which should be taken into account, as playgrounds are designed with features that engage the upper body would not be detected *via* an ambulatory monitor. The inclusive community playground used during data collection in the present study was specifically designed with structures to engage children with varying levels of physical ability. These features included, for example, ramps and a playground surface which allows easier mobility for individuals using a wheelchair/walker. By adopting an inclusive playground design, school and city planners would be addressing the needs of the whole community (17, 31, 32, 33) (United Nations Human Rights—Convention on the Rights of the Child^3^). This study demonstrates that typically developing children can achieve moderate or higher intensity exercise and HR, on an inclusive playground, which improves the likelihood that they will meet WHO guidelines for PA. Both typically developing children and those with disabilities, would benefit from a setting where they can easily interact and participate in parallel play with their peers.

In the present study, there are several limitations. The playground utilized in this study was designed for children ages 2–10 years, therefore, older children did not appear to frequent this playground as often. This was reflected in the participant demographics, with only 7 children at or above 11 years of age (Table 1). While a small sample size prevented statistical comparisons to other age groups, the results of the adolescent age group have been included in the data tables and in the supplementary Tables. The WHO has recently shown a global increase in the number of adolescent children failing to meet PA guidelines and there is a need for more research into the interest of these children in regards to playground usage.

Playground features have been shown to have a significant effect on a child's PA levels (7, 9, 12, 16, 34, 35). This study was conducted on a single inclusive community playground with specific design features to engage and include children with mobility restrictions. The current findings are limited to one inclusive playground.

Another limitation of the study was that all participants were required to wear a chest strap, sport watch and an ankle device while they played. Although no child complained or refused to participate after donning the devices, it is unclear if these multiple devices affected the participants’ typical play activity. Finally, numerous studies have shown that weather can also affect PA levels (36). To minimize this effect, all data in the current study were collected during the summer months; however, this does limit the interpretation of these results to other seasons or other regions of the country.

Further work is needed to objectively analyze the ambulatory intensity/duration and the time spent in various HR zones of children with *physical disabilities*, in this same setting. In addition, further analysis can be conducted investigating the inclusive playground features usage and their relation to elevated physical activity levels. Data presented in the current study serves as a baseline analysis that can be used for comparison purposes.

## Data Availability

The raw data supporting the conclusions of this article will be made available by the authors, without undue reservation.

## Footnotes

^1^World Health Organization Physical activity fact sheet: *World Health Organization*; https://www.who.int/dietphysicalactivity/factsheet_young_people/en/.

^2^Centers for Disease Control and Prevention Target Heart Rate and Estimated Maximum Heart Rate: *Centers for Disease Control and Prevention*. https://www.cdc.gov/physicalactivity/basics/measuring/heartrate.htm.

^3^United Nations Human Rights – Office of the High Commissioner: Convention on the Rights of the Child – Article 31 https://www.ohchr.org/en/instruments-mechanisms/instruments/convention-rights-child#:∼:text=Article%2031,-1.&text=States%20Parties%20shall%20respect%20and%20promote%20the%20right%20of%20the,artistic%2C%20recreational%20and%20leisure%20activity.
