# Supplementary material for: Inclusive community playgrounds benefit typically developing children: An objective analysis of physical activity
Source: Front Sports Act Living. 2023 Feb 1;4:1100574. doi: 10.3389/fspor.2022.1100574 (PMC9929159; doi:10.3389/fspor.2022.1100574)
Supplement: Supplementary file 1 [file Table1.docx]

Supplementary Table 1. *11+ yo* participants provided as a supplementary table; Sex comparisons – Overall Play Measures, Ambulatory and Heart Rate Measures (Mean ± SD)

|  | *Males* (n=4) | *Females* (n=3) |  |  |  |  |
| --- | --- | --- | --- | --- | --- | --- |
| Total Time (min.) | 30.4±7.1 | 49.0±21.2 |  |  |  |  |
| TAT (min.) | 25.1±4.2 | 35.9±20.9 |  |  |  |  |
| Steps | 1945±244 | 2386±1422 |  |  |  |  |
| Distance (m) | 788±236 | 579±224 |  |  |  |  |
| % of Recommend Steps | 17.7±2.2 | 21.1±12.9 |  |  |  |  |
| MVPA (min.)^1^ | 30.5±7.2 | 49.0±21.0 |  |  |  |  |
| Ambulatory Activity^2^ | | | | | | |
| *Easy* (%) | 31.1±20.8 | 63.3±33.3 |  |  |  |  |
| *Moderate+* (%) | 68.9±20.8 | 36.7±33.3 |  |  |  |  |
| *Short* (%) | 26.3±15.4 | 53.1±24.4 |  |  |  |  |
| *Intermediate* (%) | 50.0±17.5 | 21.8±18.9 |  |  |  |  |
| *Long* (%) | 23.7±28.7 | 25.1±22.3 |  |  |  |  |
| *Easy/Short* (%) | 12.5±8.0 | 35.2±23.1 |  |  |  |  |
| *Easy/Intermediate* (%) | 18.5±15.2 | 6.9±11.9 |  |  |  |  |
| *Easy/Long* (%) | 0.0±0.0 | 21.2±18.4 |  |  |  |  |
| *Moderate+/Short* (%) | 13.7±9.4 | 17.9±18.6 |  |  |  |  |
| *Moderate+/Intermediate* (%) | 31.5±27.1 | 15.0±16.7 |  |  |  |  |
| *Moderate+/Long* (%) | 23.7±28.7 | 3.9±6.7 |  |  |  |  |
| Heart Rate^3^ |  |  |  |  |  |  |
| *HR Easy* (%) | 0.0±0.0 | 0.3±0.4 |  |  |  |  |
| *HR Moderate* (%) | 10.0±12.4 | 22.0±10.0 |  |  |  |  |
| *HR Vigorous* (%) | 48.4±21.3 | 61.2±21.0 |  |  |  |  |
| *HR Peak* (%) | 41.6±25.1 | 16.6±16.5 |  |  |  |  |
| *HR Easy* (min.) | 0.0±0.0 | 0.2±0.3 |  |  |  |  |
| *HR Moderate* (min.) | 2.5±2.4 | 10.5±6.6 |  |  |  |  |
| *HR Vigorous* (min.) | 14.8±7.5 | 32.7±22.4 |  |  |  |  |
| *HR Peak* (min.) | 13.2±9.0 | 5.8±3.6 |  |  |  |  |

^1^ MVPA – moderate-to-vigorous (including peak) physical activity reported in minutes;

^2^ Ambulatory Intensity Levels and Duration periods along with the combination of Intensity/Duration reported as a percentage of total ambulatory time (TAT)

^3^ Heart Rate (HR) zones reported as a percentage of time and reported in number of minutes in each zone
