# Supplementary material for: Inclusive community playgrounds benefit typically developing children: An objective analysis of physical activity
Source: Front Sports Act Living. 2023 Feb 1;4:1100574. doi: 10.3389/fspor.2022.1100574 (PMC9929159; doi:10.3389/fspor.2022.1100574)
Supplement: Supplementary file 2 [file Table2.docx]

Supplementary Table 2. Sex comparisons across age groups (*4-6 yo* and *7-10 yo*)^1^ – Overall Play Measures, Ambulatory Activity, and Heart Rate (Mean ± SD; **p*-value <0.05)

|  | *Males* | | *p - value* | *Females* | | *p - value* |
| --- | --- | --- | --- | --- | --- | --- |
|  | *4-6 yo* (n=24) | *7-10 yo* (n=20) |  | *4-6 yo* (n=17) | *7-10 yo* (n=27) |  |
| Total Time (min.) | 30.0±12.9 | 37.9±19.6 | 0.43 | 32.3±12.9 | 27.0±10.9 | 0.18 |
| TAT (min.) | 23.5±10.4 | 30.4±15.7 | 0.33 | 26.9±11.1 | 23.1±9.7 | 0.31 |
| Steps | 1665±762 | 2099±978 | 0.21 | 1915±799 | 1629±721 | 0.26 |
| Distance (m) | 482±317 | 607±327 | 0.20 | 540±283 | 462±251 | 0.46 |
| % of Recommend Steps | 15.1±6.9 | 19.1±8.9 | 0.21 | 17.4±7.3 | 14.8±6.6 | 0.26 |
| MVPA (min.)^2^ | 29.9±12.8 | 37.9±19.7 | 0.45 | 31.9±13.0 | 27.0±10.8 | 0.25 |
| Ambulatory Activity^3^ | | | | | | |
| ***Easy* (%)** | 83.8±20.4 | 74.9±26.6 | 0.21 | **87.6±18.3** | **73.8±23.3** | **0.01*** |
| ***Moderate+* (%)** | 16.2±20.4 | 25.1±26.6 | 0.21 | **12.4±18.3** | **26.2±23.3** | **0.01*** |
| *Short* (%) | 38.2±19.2 | 30.7±21.3 | 0.19 | 28.0±15.9 | 30.7±16.5 | 0.49 |
| *Intermediate* (%) | 37.9±20.0 | 33.2±13.8 | 0.25 | 46.6±25.9 | 45.1±19.3 | 0.98 |
| *Long* (%) | 23.8±28.9 | 36.0±28.2 | 0.09 | 25.4±29.8 | 24.2±21.2 | 0.94 |
| *Easy/Short* (%) | 33.3±15.5 | 25.1±19.3 | 0.06 | 24.1±14.5 | 25.2±17.4 | 0.84 |
| *Easy/Intermediate* (%) | 33.4±20.9 | 23.2±15.2 | 0.06 | 38.1±24.5 | 34.1±20.9 | 0.69 |
| *Easy/Long* (%) | 17.0±22.7 | 26.6±28.9 | 0.18 | 25.4±29.8 | 14.6±20.0 | 0.19 |
| *Moderate+/Short* (%) | 4.9±8.4 | 5.6±5.8 | 0.31 | 3.9±4.7 | 5.5±4.4 | 0.17 |
| ***Moderate+/Intermediate* (%)** | **4.5±10.6** | **10.0±12.9** | **0.02*** | 8.5±18.0 | 11.0±11.7 | 0.09 |
| ***Moderate+/Long* (%)** | 6.8±19.9 | 9.5±20.2 | 0.51 | **0.0±0.0** | **9.7±16.4** | **0.01*** |
| Heart Rate^4^ |  |  |  |  |  |  |
| *HR Easy* (%) | 0.7±2.6 | 0.6±2.0 | 0.75 | 1.8±6.0 | 0.5±1.1 | 0.61 |
| *HR Moderate* (%) | 25.7±22.2 | 35.6±23.3 | 0.11 | 22.4±19.6 | 27.3±22.0 | 0.59 |
| *HR Vigorous* (%) | 51.8±16.6 | 46.3±16.8 | 0.37 | 53.5±16.9 | 52.7±15.0 | 0.66 |
| *HR Peak* (%) | 21.9±19.9 | 17.5±19.8 | 0.36 | 22.2±18.8 | 19.5±17.3 | 0.91 |
| *HR Easy* (min.) | 0.3±1.1 | 0.2±0.5 | 0.78 | 0.5±1.6 | 0.2±0.4 | 0.61 |
| ***HR Moderate* (min.)** | **7.0±6.5** | **14.7±12.9** | **0.05*** | 7.8±7.6 | 7.8±7.6 | 0.90 |
| *HR Vigorous* (min.) | 16.2±11.2 | 17.7±12.3 | 0.91 | 17.4±8.2 | 14.2±6.5 | 0.11 |
| *HR Peak* (min.) | 6.7±6.2 | 5.4±5.4 | 0.53 | 6.7±6.6 | 5.0±4.1 | 0.84 |

^1^*11+ yo* participants have been provided as a supplementary table

^2^MVPA – moderate-to-vigorous (including peak) physical activity reported in minutes

^3^Ambulatory Intensity Levels and Duration periods along with the combination of Intensity/Duration reported as a percentage of total ambulatory time (TAT)

^4^Heart Rate (HR) zones reported as a percentage of time and reported in number of minutes in each zone
