# Supplementary material for: Inclusive community playgrounds benefit typically developing children: An objective analysis of physical activity
Source: Front Sports Act Living. 2023 Feb 1;4:1100574. doi: 10.3389/fspor.2022.1100574 (PMC9929159; doi:10.3389/fspor.2022.1100574)

Supplementary Figure 1a & 1b. *11+ yo* participants distribution has been provided as a supplementary figure as they were excluded from the statistical analysis; a) Intensity levels (%): *Easy*, *Moderate+*; reported as a percentage of total ambulatory time; b) Duration periods (%): *Short*, *Intermediate* and *Long*; reported as a percentage of total ambulatory time


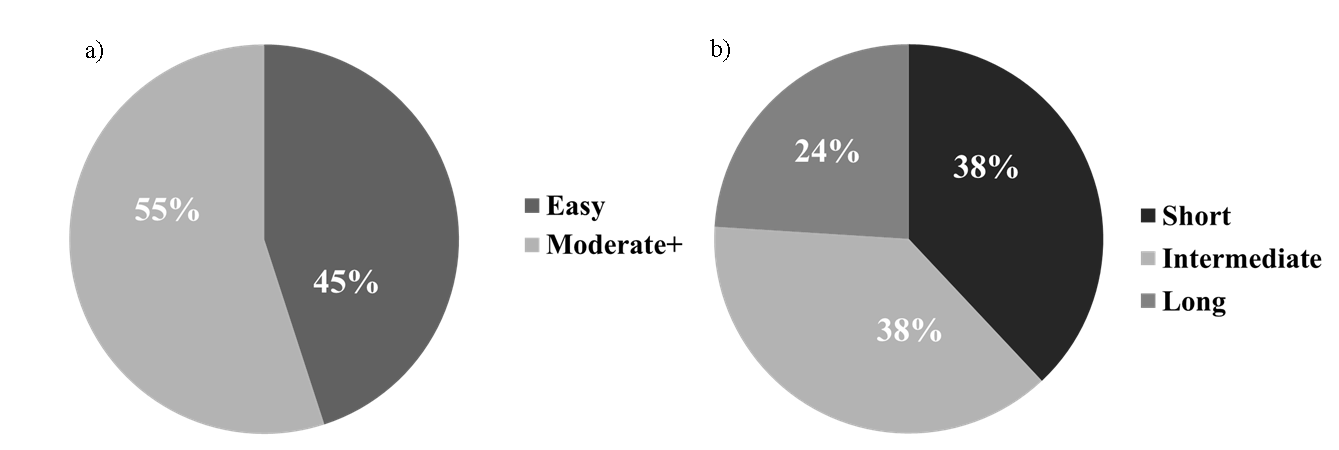

Supplement: Supplementary file 3 [file Table3.docx]
