# Supplementary material for: Inclusive community playgrounds benefit typically developing children: An objective analysis of physical activity
Source: Front Sports Act Living. 2023 Feb 1;4:1100574. doi: 10.3389/fspor.2022.1100574 (PMC9929159; doi:10.3389/fspor.2022.1100574)

Supplementary Figure 2a & 2b. a) Intensity levels (%): Easy, Moderate+; reported as a percentage of total ambulatory time; b) Duration periods (%): Short, Intermediate, and Long; reported as a percentage of total ambulatory time; 4-6 yo vs. 7-10 yo participants; 11+ yo participants distribution has been provided as a supplementary figure as they were excluded from the statistical analysis; **p*-value<0.05


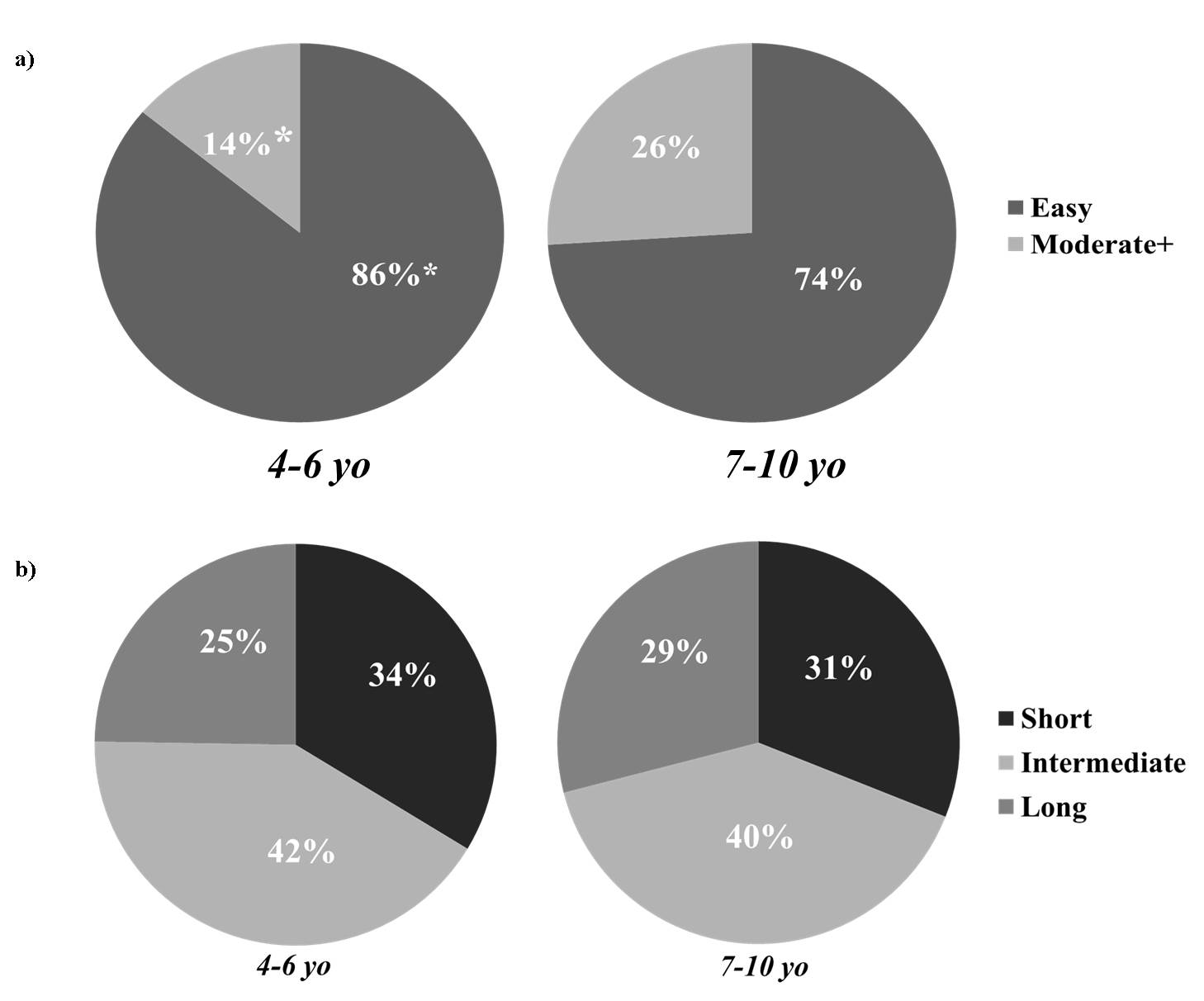

Supplement: Supplementary file 4 [file Table4.docx]
