# Supplementary material for: Inclusive community playgrounds benefit typically developing children: An objective analysis of physical activity
Source: Front Sports Act Living. 2023 Feb 1;4:1100574. doi: 10.3389/fspor.2022.1100574 (PMC9929159; doi:10.3389/fspor.2022.1100574)
Supplement: Supplementary file 5 [file Table5.docx]

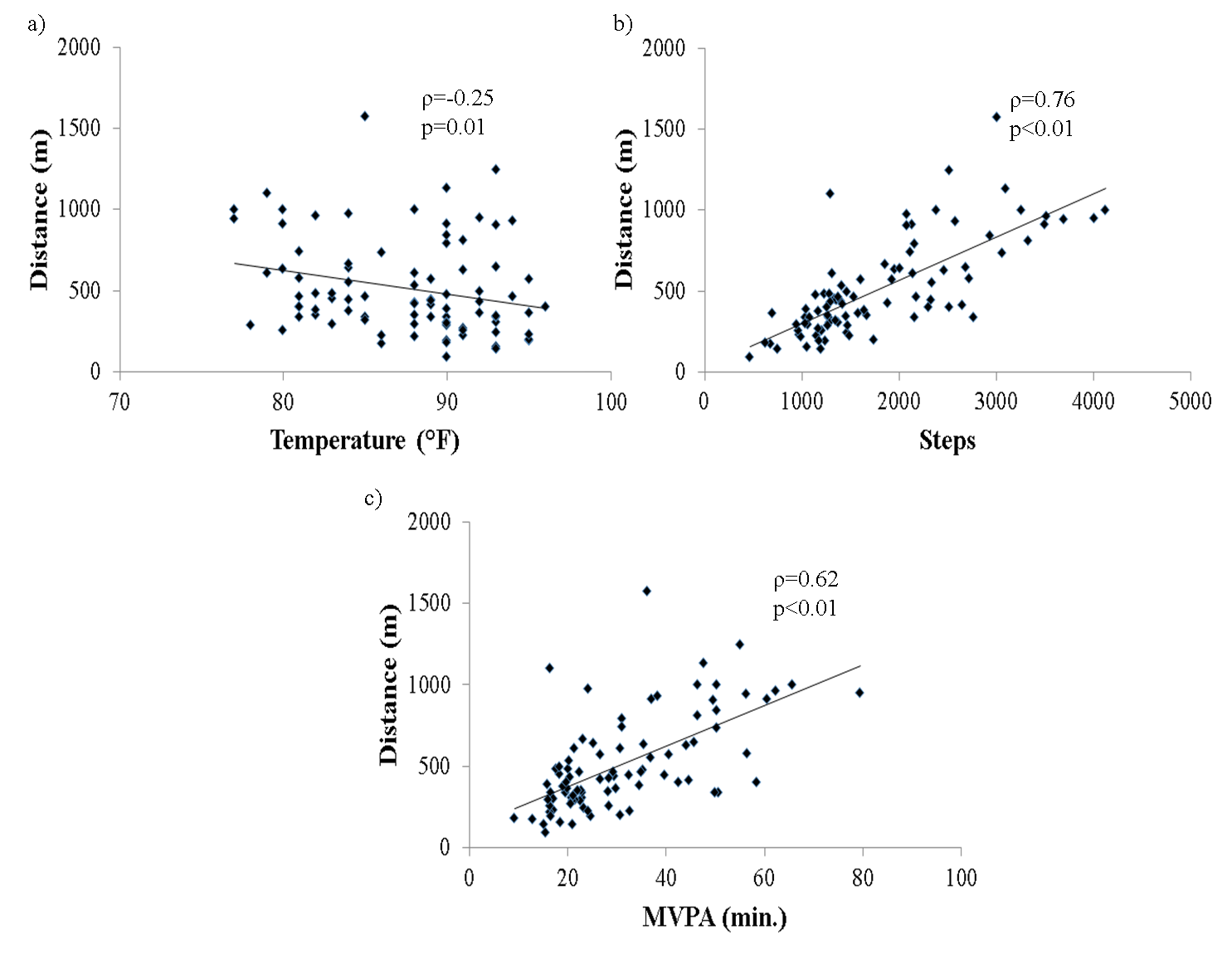
Supplementary Figure 3a-3c. Spearman rank correlations (ρ) between total distance (Distance, meters) travelled and a) temperature (°F), b) total number of steps taken (Steps), c) moderate-to-vigorous (including peak) physical activity (MVPA, minutes); all correlations were run including *4-6 yo*  and *7-10 yo* participants only. *p*-value <0.05
