# Supplementary material for: Inclusive community playgrounds benefit typically developing children: An objective analysis of physical activity
Source: Front Sports Act Living. 2023 Feb 1;4:1100574. doi: 10.3389/fspor.2022.1100574 (PMC9929159; doi:10.3389/fspor.2022.1100574)

Supplementary Figure 4a-4e. Spearman rank correlations (ρ) between participant age in years (Age, yrs.) and *Intensity/Duration* of ambulatory activity reported as percentage of total ambulatory time (TAT) a) *Easy/Short* (% TAT), b) *Easy/Intermediate* (% TAT), c) *Moderate+/Short* (% TAT), d) *Moderate+/Intermediate* (% TAT), e) *Moderate+/Long* (% TAT); all correlations were run including *4-6 yo*  and *7-10 yo* participants only. *p*-value <0.05


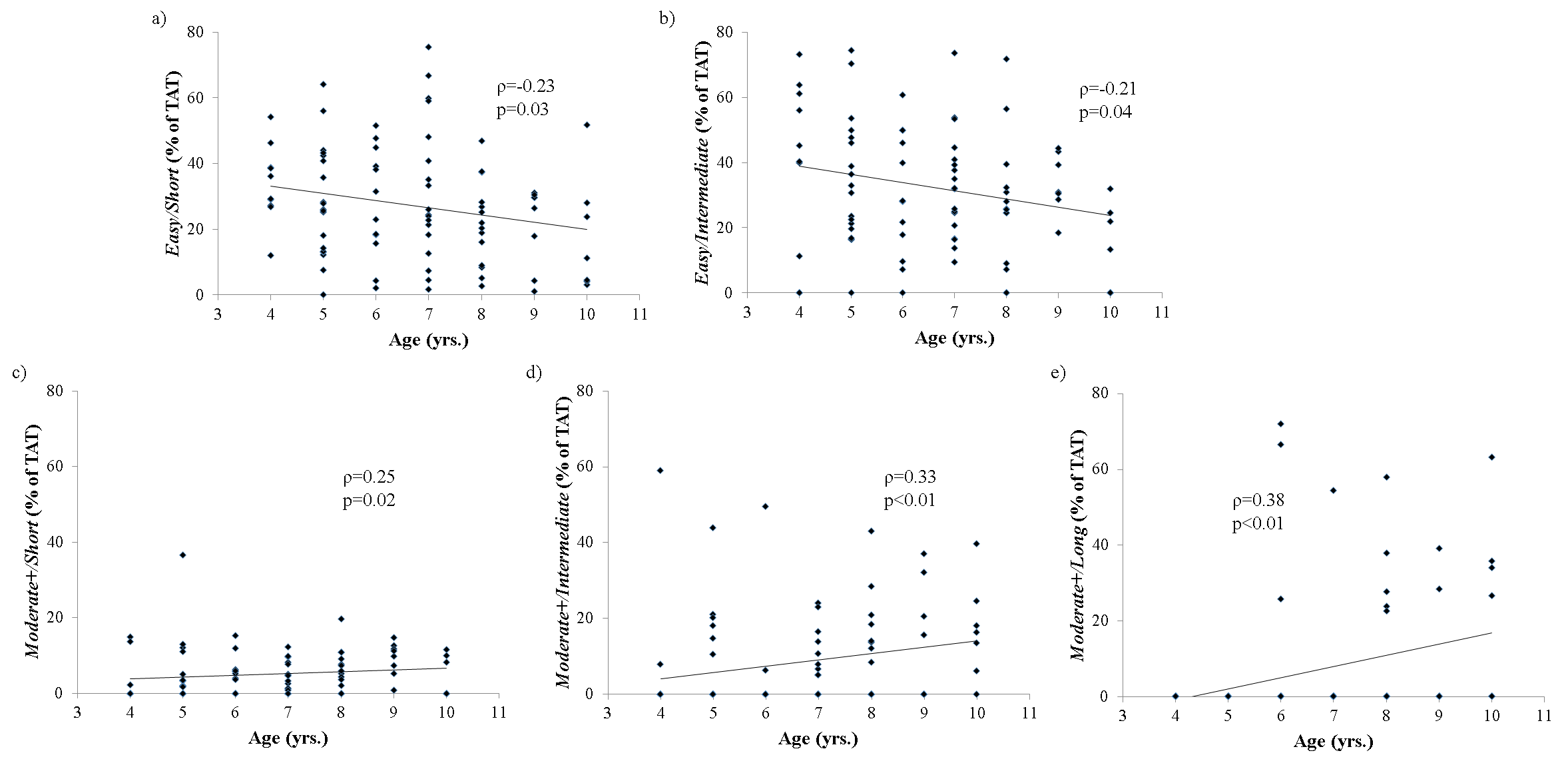

Supplement: Supplementary file 6 [file Table6.docx]
