# Supplementary material for: Inclusive community playgrounds benefit typically developing children: An objective analysis of physical activity
Source: Front Sports Act Living. 2023 Feb 1;4:1100574. doi: 10.3389/fspor.2022.1100574 (PMC9929159; doi:10.3389/fspor.2022.1100574)
Supplement: Supplementary file 7 [file Table7.docx]

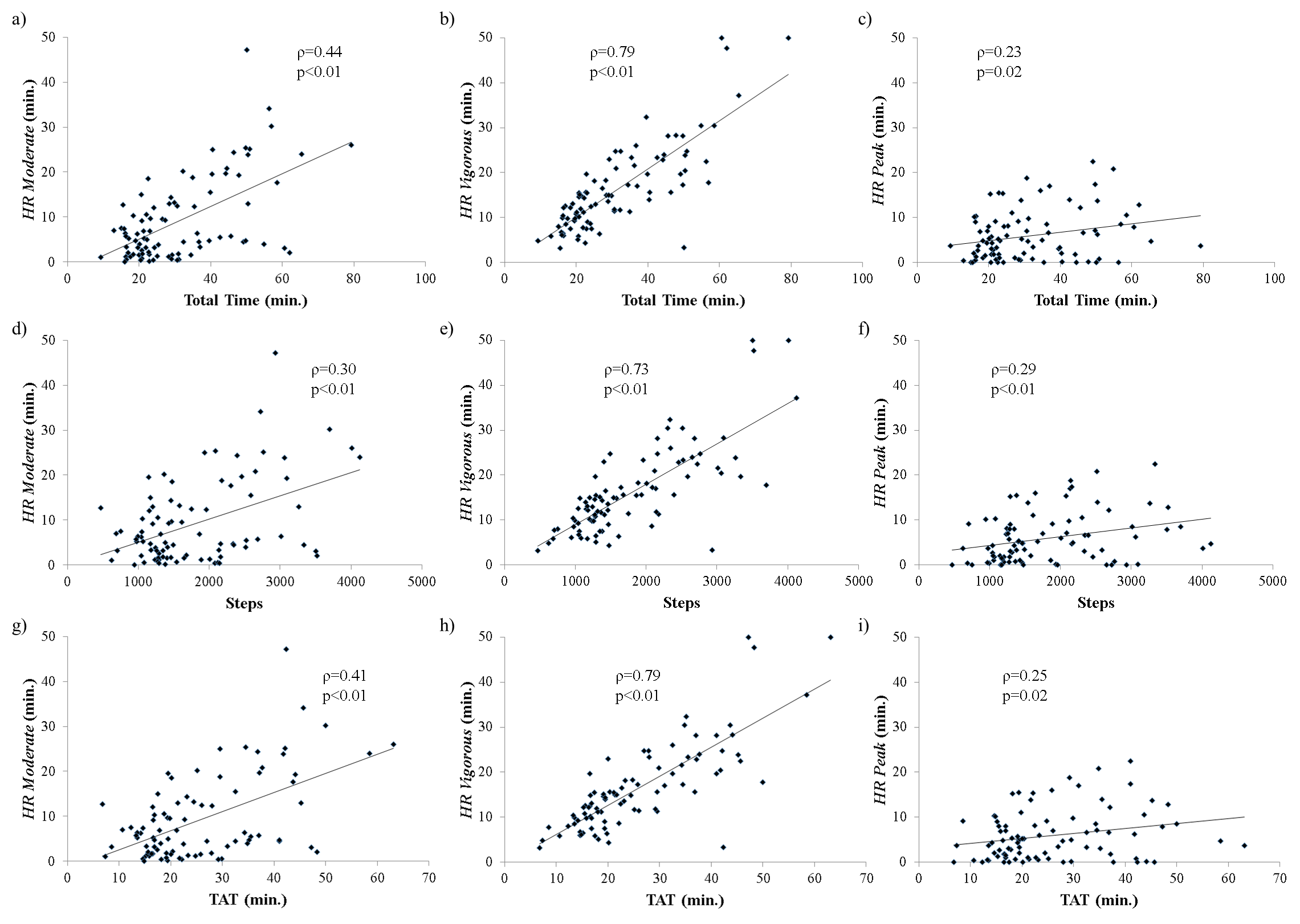
Supplementary Figure 5a-5i. Spearman rank correlations (ρ) between total time spent on the playground (Total Time, minutes) and time spent in heart rate zones (*HR*, minutes) a) *HR Moderate* (min.), b) *HR Vigorous* (min.), c) *HR Peak* (min.); total number of steps taken (Steps) and time spent in heart rate zones (*HR*, minutes) d) *HR Moderate* (min.), e) *HR Vigorous* (min.), f) *HR Peak* (min.); total ambulatory time (TAT, minutes) and time spent in heart rate zones (*HR*, minutes) g) *HR Moderate* (min.), h) *HR Vigorous* (min.), i) *HR Peak* (min.) all correlations were run including *4-6 yo*  and *7-10 yo* participants only. *p*-value <0.05
